# Conceptualising the Role of Dementia Champions Across Health and Social Care: A Qualitative Study Informed by Theory of Change (The DemChamp Study)

**DOI:** 10.1002/gps.70101

**Published:** 2025-05-21

**Authors:** Tiffeny James, Monica Leverton, Kritika Samsi, Christina Newton

**Affiliations:** ^1^ NIHR Policy Research Unit in Health and Social Care Workforce The Policy Institute, King's College London London UK; ^2^ Independent Researcher London UK

**Keywords:** dementia champion, health and social care, homecare, retention, workforce

## Abstract

**Objectives:**

People who work in health and social care frequently come into contact with people living with dementia, highlighting the need for a dementia aware and competent workforce. Some health and care services have implemented ‘Dementia Champions’ (DCs) to address this, but the role is rarely seen in domiciliary homecare services. We aim to conceptualise the DC role across health and social care to learn how it is implemented in practice and consider how it can be applied to homecare.

**Methods:**

We conducted 30 semi‐structured interviews with health and social care workers who either work as DCs or have experience/knowledge of working with them. We used framework analysis to analyse the data, informed by a Theory of Change (ToC) approach which involved identifying the ‘inputs’ involved in the role (tasks and responsibilities); and the short, medium, and long‐term mechanisms required to implement, embed, and maintain the role.

**Results:**

We identified key tasks and responsibilities of a DC which varied between and within sectors and services. The was a lack of role clarity and rarely a role description, which was considered a barrier to the role's success. The DC role is typically voluntary with no remuneration and performed on top of existing roles with no protected time for specific DC tasks. DCs typically take on the role due to a passion for good dementia care and a desire to make a difference, meaning feedback and feeling valued were important. The DC role provides an opportunity for career development, which was considered essential to retaining DCs, and health and social care workers generally. We present these findings as five themes which map onto our ToC framework to explore how the DC role is implemented, embedded, and maintained in practice.

**Conclusions:**

Across all services, there is need for role clarity, with a DC role description at the outset to set out the tasks, responsibilities, and boundaries of the role. The DC role needs protected time for workers to implement it and undertake training. We will use these findings to develop and refine our ToC framework to reflect its applicability for the homecare sector.

**Summary**

- The Dementia Champion role has been implemented in a range of health and care settings to recognise and develop staff members as dementia‐specialists. This includes hospitals and care homes (residential care), but there appear to be fewer in homecare (domiciliary care) services.
- The Dementia Champion role varies greatly across and within health and social care settings and lacks a clear role description.
- In order to successfully implement, embed, and maintain Dementia Champions in the role, protected time is needed to carry it out. However, this is rarely seen in health and social care settings which is considered a barrier to the role's success.
- Implementing the Dementia Champion role in homecare has the potential to improve quality of care for people living with dementia in their own homes and address many workforce challenges around recruitment and retention, training and development, and career progression.

## 1 Introduction

Dementia is particularly prevalent across health and social care settings. In England, approximately 70% of older people living in care homes, and 60% of those who receive care at home are people living with dementia [1]. In hospitals, 25% of acute hospital beds are used for treating people living with dementia at any time [2]. This highlights the need for a well‐trained, dementia‐aware health and social care workforce. The government set out to address this in England with the publication of its first National Dementia Strategy in 2015 [3]. The strategy, now out of date and yet to be updated, set out 17 objectives to work towards by 2020 to improve health and social care services for people living with dementia and their families in England. Objectives included having an informed and effective dementia workforce; improving the quality of care for people living with dementia in care homes; and improving quality of care for people living with dementia in general hospitals [3].

In response to this, a growing number of National Health Service (NHS) Trusts in the UK have implemented ‘Dementia Champion’ (DC) roles in hospitals. The DC role is often implemented to recognise and develop staff members as dementia‐specialists who contribute to planning and developing care provision for people living with dementia across health and care settings and promote multidisciplinary working [4]. The University of West Scotland developed a pioneering DC programme for health and care professionals working with people living with dementia being cared for in acute hospital settings in Scotland [5]. Their programme was named as one of the UK's ‘100 best breakthroughs’ for the impact it has had on people's everyday lives. As of 2021, more than 1000 people (mostly health professionals) have completed the DC programme which remains ongoing [6].

In a narrative review conducted by the research team [7], we explored the role of DCs across health and social care as evidenced in national and international literature. This work highlighted inconsistency in the use of the DC title, in the tasks and responsibilities associated with the DC role, and in service and stakeholders' understanding of the role. Findings highlighted the need for organisational and managerial support and buy‐in and ongoing professional development for DCs. Whilst DCs exist in some parts of health and social care, further research is needed to assess their impact on the quality of health and social care provision for people living with dementia; outcomes for people living with dementia and their families; staff well‐being; and cost‐effectiveness across health and social care settings. The review was part of a wider programme of work which aims to develop the role of Dementia Champions within the homecare sector. There was a notable absence of the DC role within paid homecare services, suggesting this is a sector within adult social care where DCs are least well established [7].

Most people living with dementia prefer to continue living in their own homes and can often do so with support from homecare services [8]. However, there are concerns about the quality of homecare for people living with dementia with just 2% of people living with dementia reporting that they felt homecare workers understood their needs related to dementia [9]. In their report ‘Fix Dementia Care: Homecare’, the UK's national Alzheimer's Society recommend that all homecare providers have an identified, dedicated dementia lead [9]. The DC role could go some way to addressing this. Developing a model of DCs in homecare and understanding how this can be implemented in practice may help to tackle some of the sector's challenges and improve care for people living with dementia. In this paper we build on the knowledge generated in our review of DCs across health and social care to further conceptualise the role of a DC; learn how it is implemented in practice across health and social care; and explore barriers and facilitators to implementing the role. In later phases of the study (reported elsewhere), we will discuss and consider how knowledge of the DC role across health and social care can be adapted and applied specifically to homecare for people living with dementia.

## 2 Methods

### 2.1 Ethical Approvals

Ethical approval for this study was awarded by the Health Research Authority Social Care Research Ethics Committee (ref: 23/IEC08/0011) and by King's College London University Research Ethics Board (ref: MRA‐21/22‐33445).

### 2.2 Study Design and Theoretical Framework

This qualitative study is the second phase of a four‐phase National Institute for Health and Care Research (NIHR) School for Social Care Research funded programme entitled ‘Developing the role of Dementia Champions in the homecare sector’ (the ‘DemChamp’ study). The overall aim of the DemChamp study is to define and refine the role of DCs in homecare using a Theory of Change (ToC) approach. ToC is used to understand how and why an initiative works. Part of the process involves identifying the activities of an initiative (i.e., the ‘inputs’), and the short, medium, and long‐term mechanisms of action required to achieve the initiative's intended outcome (De Silva et al., 2014). For the DC role, this means identifying the tasks and responsibilities of a DC and understanding what is required for the role to be implemented (short‐term), embedded (medium‐term), and maintained (long‐term). ToCs are developed in collaboration with stakeholders and modified throughout the development process (De Silva et al., 2014).

In our narrative review (conducted in the first phase of the study), we sought to identify the inputs/activities, and the short‐, medium‐, and long‐term mechanisms of action required for the DC role across health and social care [10] (currently under review). We developed a preliminary ToC framework based on this (see Supporting Information S1: Appendix 1). Findings from the review informed the development of interview topic guides for the qualitative work reported in this article (Phase 2) in which we aimed to further conceptualise the role of a DC; learn how it is implemented in practice across health and social care; and explore barriers and facilitators to its implementation. The need for this research was identified in previous research by the team which involved people living with dementia, family carers, and homecare workers. All aspects of this project are supported with input from our Partnership Group made up of people living with dementia, family carers, health and care professionals, and our Expert Steering Group of researchers and other relevant expert stakeholders. Both groups contributed to the development of the project by ensuring that its aims, research questions, and methods were appropriate for and could benefit people living with dementia, family carers, and homecare workers. They supported the development of participant facing study documents and interview topic guides; contributed to the interpretation of study findings; and provided input and feedback on all study outputs. The project's Research/Patient and Public Involvement Lead (CN) is a lay researcher with extensive experience in the dementia care. This paper reports findings of the qualitative Phase 2 study only.

### 2.3 Sample and Recruitment

We recruited health and social care workers across the UK who either: (1) currently or previously worked as a DC; (2) currently or previously worked alongside DCs, for example, as a colleague or manager of DCs within the same service; or (3) had other relevant experience of DCs through their work. The latter included staff from services that do not have DCs but may encounter them through their shared work with people living with dementia (e.g., Admiral Nurses), and people involved in research and training development for DCs. We used volunteer sampling by advertising study details on social media and relevant forums and networks accessible to the research team. We also identified participants purposively by searching Linked‐In and relevant health and social care websites and contacting people directly. Lastly, we used snowball sampling, asking participants to share study details within their service teams. We sent a study information sheet to anyone interested in taking part and gave participants the opportunity to ask questions before proceeding.

### 2.4 Data Collection

We conducted semi‐structured interviews between September 2022 and August 2023. We used separate topic guides for the three participant groups, with questions about the tasks and responsibilities, recruitment, training, positioning, support, and remuneration for the DCrole (see Supporting Information S2: Appendix 2). Topic guides were developed based on the Phase 1 preliminary ToC by the lead author with support from our Research/Patient and Public Involvement Lead and study Partnership Group. Participants were interviewed by telephone or video call depending on their preference. We emailed participants a consent form to complete electronically and return to us before the interview, but also offered the option to talk through the consent form verbally. For the latter, we recorded the verbal consent process and stored recordings separately to interview recordings. We collected demographic information about each participant's age, gender, and ethnicity, as well as details about their role including the sector, service type, and region of the UK they worked in. All interviews were conducted by the lead author. Interviews were audio‐recorded and transcribed by a professional company, with whom we had a confidentiality agreement.

### 2.5 Data Analysis

Interview transcripts were fidelity checked, and identifiable information was removed. We analysed data using framework analysis, following guidance by Ritchie and Spencer [10] involving 7 stages: (1) transcription; (2) familiarisation with the interview; (3) coding; (4) developing a working analytic framework; (5) applying the analytic framework (indexing); (6) charting the data into the framework matrix, and (7) interpreting the data. Framework analysis is well suited to applied, qualitative health research [11]. We developed an initial analytic framework based on topic guide questions (see Supporting Information S1: Appendix 2) and used this to code each transcript deductively. We supplemented this with open coding to identify additional codes inductively. We used the analytic framework as our framework matrix and charted data into the matrix as we coded. The lead author read and coded all interview transcripts, and 50% of transcripts were double coded by other members of the research team. The research team met throughout the coding process to discuss our ideas and add inductively identified codes to the analytic framework.

We took a realist, post‐positivist theoretical approach to the analysis and interpretation of this data. This means that as researchers, we assume there is one ‘reality’ and that this can be reported by participants and captured objectively by the researcher. However, our view is that whilst objectivity is desirable, it cannot be guaranteed as we can only understand the world and people's experiences of it from our own position [12]. This position was embedded into our study process as a whole, using a ToC (a realist approach) to guide all phases.

We used the preliminary ToC developed from Phase 1 of the DemChamp study (see Supporting Information S1: Appendix 1) to guide our analysis and interpretation of the data. This involved mapping data from the qualitative interviews onto the short‐, medium‐, and long‐term mechanisms of action required for the DC role across health and social care identified in Phase 1. The research team met throughout the analysis process to discuss preliminary ideas and interpretations which we shared and discussed with our Partnership and Steering Groups. This helped to ensure that the findings reflected what was important to the people it concerns meaning people living with dementia, family carers, and health and social care professionals, and supported our development of the final themes through an iterative process. Due to the richness of the data, we were able to generate broad themes explaining phenomena related to the DC role across health and social care, moving beyond descriptions of specific cases [11].

## 3 Findings

### 3.1 Contextual Information

We interviewed 30 participants of whom 14 (47%) were or had been DCs, 10 (33%) were working or had worked alongside DCs, and 6 (20%) were other relevant professionals with knowledge of DCs. Interviews ranged from 13 to 71 min with an average length of 36 min. Participants worked in a range of settings including acute hospital wards; private health clinics; care homes (residential care); homecare (domiciliary care); local authorities; third sector organisations; education; and research. Demographic characteristics of participants are reported in Table 1.

**TABLE 1:** Demographic characteristics of participants.

	Total (*n* = 30)
Sector—*n* (%)	
Health care	8 (27)
Social care	14 (46)
Both	8 (27)
Gender—*n* (%)	
Female	24 (80)
Male	6 (20)
Age (years)	
18–30	2 (7)
31–40	6 (20)
41–50	7 (23)
51–60	11 (37)
61–70	3 (10)
71–75	1 (3)
Ethnicity ‐ *n* (%)	
White british	20 (67)
English	15 (50)
Scottish	4 (13)
Welsh	1 (3)
White—other	2 (7)
Asian—Bangladeshi	1 (3)
Asian—pakistani	1 (3)
Asian—other	3 (10)
Black african	2 (7)
Other—mixed	1 (3)
Organisation location—*n* (%)	
England	23 (77)
London	7 (23)
South east	4 (13)
South west	1 (3)
East of england	2 (7)
East midlands	5 (17)
Yorkshire and humber	2 (7)
North east	1 (3)
North west	1 (3)
Scotland	
South eastern	1 (3)
South west	1 (3)
East central	2 (7)
Central	1 (3)
West	1 (3)
Wales	
South east wales	1 (3)

### 3.2 Tasks and Responsibilities of the DC Role (Inputs/Activities)

There was no ‘one‐size‐fits‐all’ approach to the DC role which varied greatly between and within sectors and services in terms of their tasks and responsibilities. Across all participant groups, DCs were described broadly as ‘advocates’ for dementia which involved, for example, raising dementia awareness, promoting the needs of people living with dementia, and inspiring others. The role itself ranged from DCs having just one responsibility, such as encouraging colleagues to fill in ‘This is me’ booklets with information about people living with dementia when they enter a hospital ward, to more involved roles involving the evaluation of services to identify gaps; production of an action plan; and implementation of changes to benefit people living with dementia on a hospital ward. These more involved tasks were mostly associated with the Scottish DC programme. For this, staff had a 6‐month training programme involving 5 days of face‐to‐face learning (or live online learning during COVID‐19) and a minimum of 200 h of self‐study. Training for DCs in other services ranged from a 45–60‐min dementia awareness session; to a half day training; or 8‐h across several weeks. This variation in training for DCs aligns with findings from our Phase 1 narrative review ([7]currently under review). Table 2 shows a summary of the tasks and responsibilities that DCs currently have across various health and social care services; as described by participants and grouped into four categories: (1) direct involvement with people affected by dementia; (2) direct involvement with colleagues; (3) organisational/service related; and (4) development for the DC.

**TABLE 2:** Tasks and responsibilities of Dementia Champions across health and social care.

Direct involvement with people affected by dementia	Direct involvement with colleagues
Advocate best dementia care practices Promote meaningful activities and engagement Be a named person for service users Be a link between services and families Provide tangible solutions to specific dementia related issues Signpost to other services Develop care plans for people living with dementia Quality check clients' care/care plans	Share dementia knowledge with colleagues Be a resource for colleagues to go to with queries or concerns Be a role model for and inspire other staff Deliver training to staff Support hospital ward staff with dementia related issues Supervise care home staff and activity coordinators Provide field supervision for homecare workers Encourage colleagues to fill in ‘this is me’ booklets (i.e., promote person‐centred care)
**Organisational/service related**	**Development for the DC**
Participate in service audits Identify what needs changing/improving Being an ‘agent for change’ Evaluate the impact of any changes Make wards dementia friendly Be identifiable (‘wear a pin’) Be a named person for staff e.g., being listed on the service website Raise money for dementia causes	Attend training Keep knowledge up to date Become knowledgeable on the mental capacity Act

### 3.3 Qualitative Themes

We generated five themes to further conceptualise the role of a DC and how it is implemented, embedded, and maintained in practice across health and social care. We provide quotes to demonstrate themes with contextual information about whether participants were a Dementia Champion (DC); a health and social care professional (HSP) with experience of working alongside DCs within the same service; or other professional (OP) with relevant knowledge or experience of DCs. Table 3 shows the themes and how they relate to our preliminary ToC model (see appendix 1).

**TABLE 3:** Themes and their relation to the Theory of Change (ToC) model.

ToC	Theme
Implementing (short term)	1: Becoming a DC—passion as a motivator and an essential value
	2: Incentivisation—‘money isn't everything’
Embedding (medium term)	3: Role clarity—managing expectations and boundaries
	4: Scheduling—without protected time, it's only ‘paying lip service’
Maintaining (long term)	5: Implications of and solutions to prevent high staff turnover—consistency of DCs and an opportunity to retain staff

#### 3.3.1 Theme 1: Becoming a DC—Passion as a Motivator and an Essential Value

Across some health and social care services, the DC role was initially implemented in response to the government's 2015 dementia strategy for England [3]. Recruitment approaches were varied with some services advertising the role for existing members of staff to apply for and in others, an ‘obvious’ person for the role was approached by management and asked to take on the DC role.

Participants from all groups suggested that the main motivator for taking on the role was a passion for and interest in dementia and dementia care, sometimes coming from personal experience of having a relative with dementia. Passion for the job stirred up motivation to improve knowledge to help people living with dementia:

The main motivation was the fact that I love what I do […]. So the self‐interest was there, the self‐motivation was there, and the passion was there to help an individual.

DC1

Others described an altruistic desire to help others:

I just love it, I don’t know why, I feel such a hunger for everything related to elderly, it just comes from within […]. And I feel that if I have the knowledge, I can do more, I can understand more, I can help more, so I just love it.

DC2

Passion was viewed as more important than prior dementia experience or training, with participants suggesting that as long as there is passion, knowledge can be provided:

They were all chosen because they were people with a real passion and enthusiasm for making a difference to the lives of somebody with dementia. Not necessarily that they had all the knowledge to begin with because we could provide the training.

HSP1

Passion was perceived as crucial for the role to be successful, and a core facet of the DC role:

Some people have had their names put forward for it, and it's not necessarily something they're passionate about or want to do. But it's foisted upon them, and I think in those cases it probably doesn't work that well.

OP1

The essence of [the DC role] comes from the fact that they want to do that in the first place, and it’s making sure that those people that are saying, ‘I’d like to become a champion’ aren’t being coerced by their managers because it ticks a box.

DC3

In some cases, managers approached individuals to ask them to become a DC because they were the *‘obvious person’* [HSP1] who *‘would be brilliant for the role’* [HSP4]. Others however, were ‘voluntold’ to take on the DC role, and not always for the right reasons, meaning staff were placed into the role unwillingly and without motivation. In the example below, staff were told to go on training to become a DC to improve their overall quality of work:

Some people are still voluntold, so, you know, they’re told they must go on the [training] programme, and that’s often because their practice might be quite poor, and this is seen as a way to sort them out.

HSP2

Some participants reported being named as the DC by their manager, as they were already doing tasks perceived to be associated with the role. Others were simply told that they would be the DC for the service going forward. In social care settings, managers sometimes created and took on the role themselves, filling a gap in service need.

#### 3.3.2 Theme 2: Incentivisation—‘Money Isn't Everything’

Continuing to look at the short‐term mechanisms of action to implement this role, it was important to identify what, if any, incentives were required to motivate staff to take on and stay in the role. The DC role was experienced or understood by many as a voluntary position for which DCs did not receive additional pay or reward.

For DCs who were nurses in healthcare settings, there was an argument that some of the tasks and responsibilities associated with being a DC, for example supporting and teaching colleagues, were already an expected part of the nursing role and therefore would not receive additional remuneration. In some cases, the DC was in a more senior position and therefore already receiving a higher rate of pay than other colleagues, but did not receive additional pay to be a DC. Whilst some felt that this was unacceptable…

I think that's a huge failing. And when you go back to roles and responsibilities, I think if it is formalised and if there is an expectation to meet a certain standard, then there should be some remuneration or something.

OP2

…others felt it was reasonable due to the opportunities the role provides for career progression:

There was that sort of sense of a career progression that people could get towards it through the Dementia Champions route, I would say, but they didn't get any more money for doing it.

HSP3

DCs themselves did not mention career progression as a motivator for, or benefit of taking on the role, instead discussing altruistic motivations, such as the sense of satisfaction they get from the work:

It’s not just about money, but the fact that you are being recognised, you are being appreciated, you are feeling valued for the job that you do.

DC4

It was highlighted by all participant groups that letting staff know they are doing a good job is an important way to value them:

It's showing people the value of them and what they're doing […] thanking people and telling them the good things they're doing because I think particularly care workers, often brush off what they're doing as what they always do […] and they need other people to tell them that what they're doing is fantastic.

HSP1

This resonated with the overall belief from participants that feeling valued was perhaps more important than financial reward.

#### 3.3.3 Theme 3: Role Clarity—Managing Expectations and Boundaries

Successfully embedding DCs within a service required role clarity. Participants discussed the importance of a clearly defined DC role to help the DC know what is expected of them and to manage expectations of their managers, other colleagues, people living with dementia, and family carers by knowing what they can ask of the DC. This can reduce the risk of disappointment by setting appropriate boundaries for all:

If you've got a clearly defined role at the outset, then even the most passionate or interested person knows what they're buying into, what they're offering to do. I think in terms of expectations of the role from themselves above another's, it needs to be very, very clear.

OP2

The disadvantage when it comes to Dementia Champions is if a caregiver thinks, ‘Oh, the Dementia Champion will sort things out for me’ […], so it’s just making sure you communicate [the role], especially to the caregivers, because they, I’m sure, will have a huge expectation.

DC3

When the role is unclear, there can be crossed boundaries, with DCs ‘working outside of their scope of responsibility’ [P2O1], potentially putting themselves, their colleagues, the services, and the people they support at risk:

[…] for example [the DC] taking the lead on reporting something when it should have been escalated to the most appropriate person […] and they'll be accountable for whatever outcome or impact that has.

OP2

This could create additional stress for the individual DC and impact their satisfaction with the role. Though many participants discussed the importance of a clearly defined role, this was not always done in practice. One participant felt that this could be why the DC role has not, in their view, succeeded so far:

I wonder if that's somewhere the idea falls down slightly, that it's not a well enough defined role […] maybe a lack of clarity for the [DC] about what their responsibilities are, and other people about what they can expect from [the DC].

OP1

Without a clear description, and subsequently a well‐thought‐out plan for the role, there is a risk that the DC role does not live up to its potential:

If the role’s ill‐defined then it could be a wasted opportunity. […] if the time’s been invested, if the person’s been supported to go [on training] but they’re not clear on their role and the people around them aren’t clear on their role then you do potentially have that waste of time, effort and money.

DC5

For example, many participants stated that a key aspect of the DC role is speaking with family carers. However, a manager [OP4] we spoke to recalled being asked by a DC within their service if DCs were allowed to speak to family carers. This suggests that in this case, the DC role may not have been clearly defined and that potentially, some valuable aspects of the role were not being fulfiled.

#### 3.3.4 Theme 4: Scheduling—Without Protected Time, It's Only ‘Paying Lip Service’

The DC role was typically embedded within or performed on top of existing roles, rather than being a separate, standalone role. All but one participant we interviewed experienced or understood the DC role as an ‘add on’ or an adjunct to a primary role. Participants from one care home setting viewed the role as being distinct from other care workers, as DCs were not expected to deliver care “on the floor”, but were there to support the team. In all other cases, however, the DC role was performed alongside a primary care role:

You’re not there to provide only Dementia Champion, that is only one percent of what we do, and we have to cover more than that.

DC2

Participants working in care settings in England described how DCs generally did not have protected time to fulfil the additional tasks and responsibilities of the DC role and instead were required to do this on top of their normal role:

So I do the Dementia [Champion role], I work with the tissue viability nurse, too. And I work with medicine management now. I do those three things on top of my role. Sometimes it's so busy that I forget that those are my roles.

DC6

In the Scottish programme, there was an expectation that DCs would be given protected time to do the role, including for training, but this did not always happen in practice:

By sending people on the programme, the senior sponsors have made a commitment to release and support the staff to do this […] that’s not how it pans out always because I think there’s a gap between senior sponsors and the line managers or the team managers, some champions will come in their own time.

HSP2

With workers juggling many competing responsibilities, the DC role becomes less of a priority, and the responsibilities of the role were deemed less important than the responsibilities of the worker's primary role:

When things are occurring that are high pressured things, or there’s not as many staff, [the DC role] can go on the backburner.

HSP4

In most cases, participants discussed a lack of staff and resources as a barrier to ensuring protected time for the DC role:

There probably is staff who have had [DC] training, but simply don’t have enough time to do anything about it, because the wards are so understaffed, people are so stretched.

DC7

In contrast, some participants highlighted potential organisational and logistical challenges arising from having protected time. One participant gave an example of how protected time for DCs would challenge the nature of domiciliary homecare:

I would say it’s down to pressure of time. I think especially homecare, they are absolutely flat out and if someone isn’t at work, there’s that gap, you know, perhaps even eight people a day aren’t getting their visit from that worker, so it is very, very difficult.

OP3

It was recognised that staff shortages can limit others' professional development, and that a lack of protected time can limit the role's potential and the satisfaction that DCs can get from it:

When you don’t have enough staff, there is a restriction in people’s capacity and ability to develop and grow.

HSP2

Something that's quite demotivating is if you get put into a role like that and find that actually, you’ve not got the support to make changes to make things matter […] so making sure you can actually do what you set out to do and feel like you've achieved something, I think is a big motivating factor for helping people stay in roles like that.

OP1

#### 3.3.5 Theme 5: Implications of and Solutions to Prevent High Staff Turnover—Consistency of DCs and an Opportunity to Retain Staff

When considering maintaining the DC role in the long‐term, participants across all settings and locations discussed high turnover as a challenge. High staff turnover in health and social care organisations overall means that there is also a high turnover of DCs in settings that have implemented them:

The turnover in those staff groups is so huge that that, from my perspective and experience in my support of Dementia Champions, you have constant changing workforce.

HSP5

This can make it unviable for organisations to maintain DCs and reduces motivations to try again, particularly when time and money has gone into training.

An organisation might have put a little bit of time and money into training them up, and then they leave to go somewhere else. And then you're sort of faced with, do we train somebody else up, or do we just scrap that role?

OP1

In larger organisations, staff turnover can make it challenging to keep track of who is a DC, and has implications for quality and consistency of care for people affected by dementia, as well as the staff supporting them:

Keeping an accurate register of the Dementia Champions is an incredible task […]. There’s something about the coordination, long‐term coordination of Dementia Champions that has proved quite difficult.

OP3

However, to improve staff turnover more generally it was suggested that opportunities for career progression, such as those provided by the DC role, could improve job satisfaction and help with staff retention:

Retentions are really tricky in both nursing and in social care […] but I think in order to incentivise and maintain someone's motivation, they need to know where they're going with the job. […] the opportunity to bring yourself up to speed through training and knowledge can be a great motivator for individuals.

OP2

Some, however, felt that training and development opportunities can result in increased staff turnover in care settings, by making DCs more employable and attractive to other sectors and organisations who can perhaps provide greater rewards or incentives. This was described as a potential disadvantage of the DC role, and of upskilling the care workforce generally:

If you train people up and give them lots of extra skills and knowledge, then the risk is, having done all that, they then become attractive to another organisation and they disappear off.

HSP1

Though DC participants did not discuss retention explicitly, some mentioned factors which would improve the role such as having more opportunities to improve skills and knowledge (training), and opportunities to get together with other DCs to *‘allow you to continue being inspired’* DC8.

## 4 Discussion

This work aimed to conceptualise the role of a DC and understand how it is implemented, embedded, and maintained in practice across health and social care. Reflecting and building on findings from our earlier narrative review [7], we found that the role varies greatly between and within settings in terms of the tasks and responsibilities DCs have. Apart from services involved in the Scottish DC programme, there was no consistency within settings or services in the role of the DC, with individual services/settings determining their own approach and understanding of what a DC is and does. How and why DCs got into the role varied, but a common reason across settings for taking on the role was a passion for good dementia care. When unmotivated or unwilling staff members were placed into the role by managers, this was seen as a failure of the role. The DC role is often poorly defined which was also perceived as a barrier to its success. Typically done in a voluntary capacity without additional pay, the DC role is seen as an ‘add on’ to existing roles, often with no protected time for implementation or training which was highlighted as another major challenge. These findings reflect and build on those from our Phase 1 narrative review [7]. We discuss our findings through a Theory of Change (ToC) lens, highlighting the short‐, medium‐, and long‐term mechanisms of action required for the DC role across health and social care.

### 4.1 Mechanisms of Action to Support the DC Role (Theory of Change)

#### 4.1.1 Short Term (Implementing the DC Role)

Across all settings, passion for good dementia care was perceived as the main motivator for someone to take on the DC role. Passion was also considered crucial for the role's success and should be considered as a core value in the DC recruitment process. This mirrors findings from our narrative review which highlighted the need to select the ‘right’ person [7]. This is also in line with recommendations by Skills for Care, the strategic workforce development and planning body for adult social care in England, who found that using values‐based recruitment approaches leads to lower staff turnover and better staff performance [13].

We found that the DC role was typically carried out on a voluntary basis (i.e., with no additional pay), taken up for altruistic reasons like wanting to make a difference to peoples' lives, rather than for financial reward. This reflects findings from existing research in which care workers supporting people living with dementia discussed a sense of pride, self‐esteem, and job satisfaction from ‘going the extra mile’ for the people they care for [14]. However, the additional voluntary labour involved in going above and beyond can also come with practical, emotional, and financial costs to care workers [14, 15] and ultimately, the exploitation of their good will. This also highlights a wider issue underlying all care work; that it is undervalued and underpaid [16]. Whilst passion is certainly a desirable quality, it is not enough for the success of the role which needs to be adequately resourced and supported [17]. This is supported by findings from our narrative review which highlighted requirements beyond the skill and passion of care workers, such as the need for organisational commitment including buy‐in from the organisation and managers; efforts to fully embed the role into the work culture; and consideration at the start of how the role can be developed and maintained including what training managers may need to support DCs [7].

#### 4.1.2 Medium Term (Embedding the DC Role)

A lack of protected time, often due to limited resources and low staffing levels, was discussed as a major barrier to embedding the DC role. This is not surprising given the huge challenge with recruitment and retention currently facing the health and social care sector [18]. As outlined above, DCs were motivated and felt rewarded by the satisfaction they got from helping individuals affected by dementia. However, without protected time, they are unlikely to be able to make the difference they seek to, leading to unmet job expectations and poor satisfaction in the role, echoing findings linking job satisfaction and work well‐being [19, 20].

#### 4.1.3 Long Term (Maintaining the DC Role)

Not being able to meet the expected aims or goals of the role can result in DCs becoming demotivated, and potentially leaving the service or giving up the DC role. In the Scottish DC programme, only staff who had the authority or seniority to be ‘agents of change’ within a service that is, those who could implement changes and improvements around dementia, were offered the DC training programme. This likely contributes to the success of this programme. Feeling valued in the role comes from knowing that they are making a difference. DCs therefore require feedback and opportunities for supervision and reflective practice. Reward and recognition have been identified as important for retention of care staff by Skills for Care [21]. Feedback can come through from managers, colleagues, people living with dementia, and their families. In some cases, we found that feedback was considered more important than financial remuneration which again, could lead to the risk of exploitation of care workers' good will.

**FIGURE 1:**
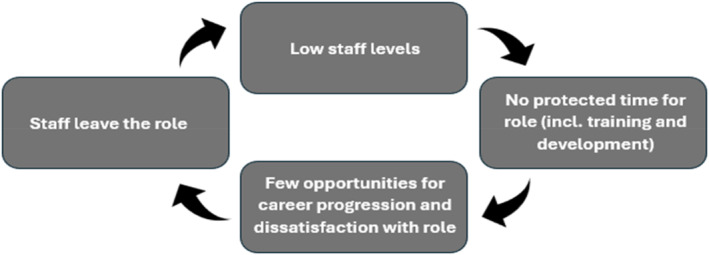
Cyclical issue of staff retention in health and social care.

There was rarely protected time given for training and development, limiting DCs' opportunities for career progression, and potentially contributing to staff leaving the role/service. This supports findings from our narrative review [7] and from NHS and Adult Social Care reports in the UK which highlight opportunities for career progression as a key motivator for potential candidates in health and social care [21, 22]. These reports recommend provision of such opportunities as a strategy to improve retention across the sector. Protected time for training is a central tenet of the Scottish programme which could explain some of its success however, participants involved in this programme discussed that this does not always happen due to differences in the priorities or understandings of senior DC sponsors and DCs' line managers. Whilst the DC role could provide an opportunity for career progression and therefore retention of staff, there will likely need to be other approaches to improve recruitment and staff levels initially due to the cyclical nature of these issues (see Figure 1).

### 4.2 A Cross‐Cutting Mechanism—Role Clarity

In our narrative review ([7]currently under review), role clarity was identified as a medium‐term mechanism of action required to embed the DC within their role and service however, findings from this qualitative study revealed that role clarity is important and relevant across all mechanisms of action that is for the implementation (short‐term), embeddedness (medium‐term), and maintenance (long‐term success) of the DC role. A clearly defined role is crucial in terms of recruitment (short‐term) to ensure that services get the ‘right person’ for the role. A role description also helps manage expectations of the DC, their colleagues, and the people they support, reducing the risk of crossed boundaries and disappointment of all parties (medium‐term). If expectations do not reflect the role accurately, DCs may become demotivated, potentially leaving the role (long‐term). Similar challenges were identified in a study of Dementia Support Workers (DSWs) based in hospitals in Wales whose primary role was to promote meaningful activity and engagement with people living with dementia, optimise person‐centred care, and support families [23]. DSWs reported feeling demotivated and undervalued due to a lack of role clarity, with some being unaware of the impact of their work and whether they were doing the job correctly. As in our study, DSWs were draughted in to carry out personal care when there were staff shortages which was not part of the role, and meant that there was no time to do tasks that the role entails. Some felt that role ambiguity was the reason some DSWs left the role shortly after starting. In a study of hospital nurses, role ambiguity was associated with increased stress, job dissatisfaction, and intention to leave [24]. Furthermore, this highlights a lack of consistency in terminology of dementia‐specialist roles. Having a consistent title for such roles may help to streamline role clarity and the tasks and responsibilities associated with these roles.

Very few services in our study had a DC role description, and there appears to be no standardised approach to the DC role across health and social care settings. This reflects findings from a quantitative survey of DCs across health and social care settings in England in which only six out of 34 DC respondents had received a role description outlining the expectations of the role [25]. The Scottish DC programme was one of the few that had a clearly defined role which could account for some of its success. There is scope for the development of a baseline DC role description with core requirements, which can be adapted to suit the needs and resources of individual services. Although roles will likely vary across health and social care settings, services of the same type (e.g., home care, care homes) will likely have similar DC roles and could benefit from a general description to support the recruitment, embeddedness, and maintenance of DCs within their services. Differences may also arise given the size of services, whereby tasks may vary for services with one DC, compared to services with several.

### 4.3 Strengths and Limitations

Whilst we aimed for a diverse sample of health and social care services to recruit from, it was difficult to identify homecare providers with DCs in their service. Many of our findings are relevant for all types of health and social care settings however, it is likely that the homecare sector will have unique needs and challenges around the DC role that were not captured in this study, such as the fact that homecare workers typically work alone, rather than on a shared site as in care homes and hospital wards. However, the fact that we were only able to identify and recruit one DC from homecare supports our understanding that this is not a well‐established role within the homecare sector. Most homecare services we contacted in our recruitment efforts did not have DCs, but were interested in implementing the role, warranting further research and development of the DC role for homecare. These services have been included in a later phase of the wider DemChamp programme [26]. Moreover, we were able to speak to a range of staff from a range of settings to collate as much information as possible about the DC role including the tasks and responsibilities as well as potential barriers and facilitators to the role, which we will use to adapt the role to homecare. This helps build a clearer vision around what a DC is and does to work towards a more streamlined role across services.

## 5 Conclusion

Our findings highlight that there is no standardised approach to the DC role across health and social care settings. This is likely to be due to (and further impact) clarity around the DC role which we identified as key to its implementation, embedding, and maintenance in health and social care settings. Those who become DCs typically do so because of their passion for good dementia care. Whilst this is considered crucial to the role's success, it risks potential exploitation of the good will of health and care workers. The fact that the DC role is largely unpaid, undervalued, and rarely given protected time highlights the fundamental lack of value that society and organisations have given to dementia and to the DC role since its inception. Given the expected increase in the number of people living with dementia, this needs to change, and the importance and value of the DC should be taken seriously within health and social care by being built into governmental narratives and policy agendas. DCs need a clearly defined role, protected time to implement the role, opportunities and time for development, and reward and recognition.

## Conflicts of Interest

The authors declare no conflicts of interest.

## Supporting information

Supporting Information S1

Supporting Information S2

## Data Availability

The authors have nothing to report.
