# Supplementary material for: Conceptualising the Role of Dementia Champions Across Health and Social Care: A Qualitative Study Informed by Theory of Change (The DemChamp Study)
Source: Int J Geriatr Psychiatry. 2025 May 21;40(5):e70101. doi: 10.1002/gps.70101 (PMC12095095; doi:10.1002/gps.70101)
Supplement: Supplementary file 1 — Supporting Information S1 [file GPS-40-e70101-s002.docx]

**Appendix 1:** Preliminary Theory of Change Framework of the short, medium, and long-term mechanisms of action required for the Dementia Champion role across health and social care.

This was developed from our phase 1 narrative review (reported in Leverton et al., 2025 – currently under review) and used as a framework for qualitative data analysis and interpretation generated from the current study.

| **Mechanisms of action required for the DC role across health and social care** | | |
| --- | --- | --- |
| Short-term (implementing) | Medium-term (embedding) | Long-term (maintaining) |
| **Organisational commitment**   - Buy-in from organisation - Embed role within service culture - Secure funding for ongoing development | **Role clarity**   - Establish protected time - Form a cemented identity - Give visibility - Provide written documentation | **A wide network of support**   - Not a lone role - Ensure opportunities for peer support - Create or support DC network |
| **Role set-up**   - Establish support network - Provide sufficient training - Develop achievable action plan - Engage with specialists - Plan for higher level support - Provide opportunities for reflective practice | **Working with others**   - Establish relationships with DCs across services - Adopt a tiered approach to staffing - Ensure respect from colleagues - Being the “go-to” dementia specialist | **Organisational and managerial support**   - Regular supervision - Support to achieve goals - Upskill with visible progression - Ensure ongoing commitment following changes in leadership - Provide opportunities for ongoing development |
| **Recruitment**  *Selection*   - Select the “right” person - Self-nominated/motivated - Provide equal opportunity for all   *DC motivation*   - Career step-up/upskilling - Passion for dementia care |  | **Achieving role expectations**   - Instil a sense of agency to influence change - Adopt measurable goals and evaluate impact - Provide recognition, encouragement, and feedback |

Leverton M, James T, Samsi K, Newton T. . The role of Dementia Champions across health and social care settings: Identifying mechanisms of action using a Theory of Change approach. Under review. 2025.
