# Supplementary material for: Conceptualising the Role of Dementia Champions Across Health and Social Care: A Qualitative Study Informed by Theory of Change (The DemChamp Study)
Source: Int J Geriatr Psychiatry. 2025 May 21;40(5):e70101. doi: 10.1002/gps.70101 (PMC12095095; doi:10.1002/gps.70101)
Supplement: Supplementary file 2 — Supporting Information S2 [file GPS-40-e70101-s001.docx]

**Developing the role of Dementia Champions in homecare: Interview topic guide Dementia Champions**

**Appendix 2**: Interview topic guides

**1. Before we begin talking about Dementia Champions, what does the term “champion” mean to you?**

**2. Now we would like to understand what your role as a Dementia Champion involves. How would you summarise your role?**

a. What kind of tasks/responsibilities do you have as a Dementia Champion?

b. Is your role as a Dementia Champion in addition to other responsibilities or roles?

c. Do you have a role or job description? (ask to see if possible)

**3. Does your role as a Dementia Champion differ to other colleagues within your team/service, and if so, how?**

**4. How did you begin your Dementia Champion role?**

1. Was the position created within your existing team/service or advertised externally?

**5. What was your motivation for taking on this role?**

**6. Does your pay/salary reflect the Champion role?**

**7. Who do you work with or provide support to in your role as Dementia Champion? This could be directly or**

**indirectly.**

**8. Is there a group that you feel particularly benefits from having a Dementia Champion in the team?**

**9. Have you had training for your role as a Dementia Champion – please describe.**

1. Was this beyond what other non-dementia specialist staff receive?
2. Had you received this sort of training before your role as a dementia champion?
3. Who delivered the training/where did training come from?
4. Is there any ongoing training?
5. Is there anything you would have liked to have but did not get?

**10. Do you receive any support in your role as Dementia Champion – please describe.**

1. What support do you find helpful?
2. What support do you find unhelpful?
3. Do you have any contact with others in the same role as yourself? (e.g., peer support)
4. Is there any other support that you feel you need or would find helpful?

**11. Are you satisfied in your role as a Dementia Champion?**

1. What, if anything, do you like about being a Dementia Champion?
2. Is there anything you dislike about the role?
3. Is there anything you find difficult about the role?
4. Is the role as you expected it would be? If no, what’s different?

**12. What would make the Dementia Champions role better?**

1. For you
2. For people living with dementia and family carers

**13. How does being a Dementia Champion factor into your future career plan?**

*For staff who previously worked as a Dementia Champion (but have since stopped the role):*

**14. Why did your role as a Dementia Champion end?**

*Depending on the answer*

1. Is there anything that would have encouraged you to stay in the role?
2. Would you have liked to continue the role if it was possible?

**Developing the role of Dementia Champions in homecare: Interview topic guide Staff working with DCs**

1. **Before we begin talking about Dementia Champions, what does the term “champion” mean to you?**
2. **Now, can you tell me in what ways you work with staff who are Dementia Champions?**
   1. Where do you work? i.e., are there DCs in your service or do you work with them in other services
3. **(For managers) Why was the role created within your team/service?**
4. **(For managers) How did the Dementia Champion get appointed to that role?**
5. **In your own words, what do you see as being the role of Dementia Champions in your team/service?**
   1. What responsibilities do they have?
   2. What tasks do they carry out?
   3. Is there a job description?
   4. How does their work differ to other care staff in the team/service?
6. **Who do Dementia Champion work with or provide support to? This could be directly or indirectly.**
7. **Is the Dementia Champion role something you would consider?**
   1. Why is that?
   2. Is there anything that would encourage you to become a Dementia Champion?
   3. Is there anything that puts you off becoming a Dementia Champion?
8. **Are there any sources of overlap or tension between the Dementia Champion’s role and anyone else’s in the team?**
9. **(For managers/trainers) What training (if any) do Dementia Champions receive?**
   1. Do they receive training beyond what other non-dementia specialist staff receive? Please describe.
10. **(For managers) What support is available for staff in Dementia Champion roles?**
    1. Does this differ to support other non-dementia specialist staff receive? Please describe.
11. **(For managers) If known, does the pay/salary reflect the Champion role? (not including for managers)?**
12. **Please describe what, if any, benefits there are of having Dementia Champions in your team/service to:**
    1. For members of staff
    2. For people living with dementia and their families
13. **Please describe what, if any, disadvantages there are of having Dementia Champions in your team/service:**
    1. For members of staff
    2. For people living with dementia and their families

*For staff in teams/services that previously employed a Dementia Champion (but this has now ended)*

1. **Why did the role of Dementia Champion in your team end?**

**Developing the role of Dementia Champions in homecare: Interview topic guide Staff with other relevant experience of DCs through their work**

1. **Before we begin talking about Dementia Champions, what does the term “champion” mean to you?**
2. **In your own words, what do you see as being the role of Dementia Champions in health and social care?**
   1. What responsibilities do they have?
   2. What tasks do they carry out?
   3. Is there a job description?
   4. How does their work differ to other care staff in the team/service?
3. **Now, can you tell me what your experience is of working with Dementia Champions across health and social care services**
   1. Are there dementia champions in your team?
   2. Does your work overlap with the work of Dementia Champions in any way?
4. **From your experience, who do Dementia Champion work with or provide support to? This could be directly or indirectly.**
5. **Are you aware of any sources of overlap or tension between the Dementia Champion’s role and any other staff across health and social care** (Probe if yes: why, what happened, how was it resolved…)
6. **We would like to know about training and support available to Dementia Champions.**
   1. Are you aware of any training that dementia champions receive?
   2. Are you aware of support that is available for staff in Dementia Champion roles?
7. **Please describe what, if any, benefits there are of having Dementia Champions in health and social care services to:**
   1. For members of staff
   2. For people living with dementia and their families
8. **Please describe what, if any, disadvantages there are of having Dementia Champions in your team/service:**
   1. For members of staff
   2. For people living with dementia and their families

**Is there anything else you would like to discuss?**
